# Characterization of Two 20kDa-Cement Protein (cp20k) Homologues in *Amphibalanus amphitrite*

**DOI:** 10.1371/journal.pone.0064130

**Published:** 2013-05-22

**Authors:** Li-Sheng He, Gen Zhang, Pei-Yuan Qian

**Affiliations:** KAUST Global Collaborative Research Program, Division of Life Science, Hong Kong University of Science and Technology, Clear Water Bay, Kowloon, Hong Kong SAR, China; University of South Florida College of Medicine, United States of America

## Abstract

The barnacle, *Amphibalanus amphitrite*, is a common marine fouling organism. Understanding the mechanism of barnacle adhesion will be helpful in resolving the fouling problem. Barnacle cement is thought to play a key role in barnacle attachment. Although several adult barnacle cement proteins have been identified in *Megabalanus rosa*, little is known about their function in barnacle settlement. In this study, two homologous 20k-cement proteins (cp20k) in *Amphibalanus amphitrite*, named Bamcp20k-1 and Bamcp20k-2, were characterized. The two homologues share primary sequence structure with proteins from other species including *Megabalanus rosa* and *Fistulobalanus albicostatus*. The conserved structure included repeated Cys domains and abundant charged amino acids, such as histidine. In this study we demonstrated that Bamcp20k-1 localized at the α secretory cells in the cyprid cement gland, while Bamcp20k-2 localized to the β secretory cells. The differential localizations suggest differential regulation for secretion from the secretory cells. Both Bamcp20k-1 and Bamcp20k-2 from cyprids dissolved in PBS. However, adult Bamcp20k-2, which was dominant in the basal shell of adult barnacles, was largely insoluble in PBS. Solubility increased in the presence of the reducing reagent Dithiothreitol (DTT), suggesting that the formation of disulfide bonds plays a role in Bamcp20k-2 function. In comparison, Bamcp20k-1, which was enriched in soft tissue, could not be easily detected in the shell and base by Western blot and easily dissolved in PBS. These differential solubilities and localizations indicate that Bamcp20k-1 and Bamcp20k-2 have distinct functions in barnacle cementing.

## Introduction

**Figure 1:**
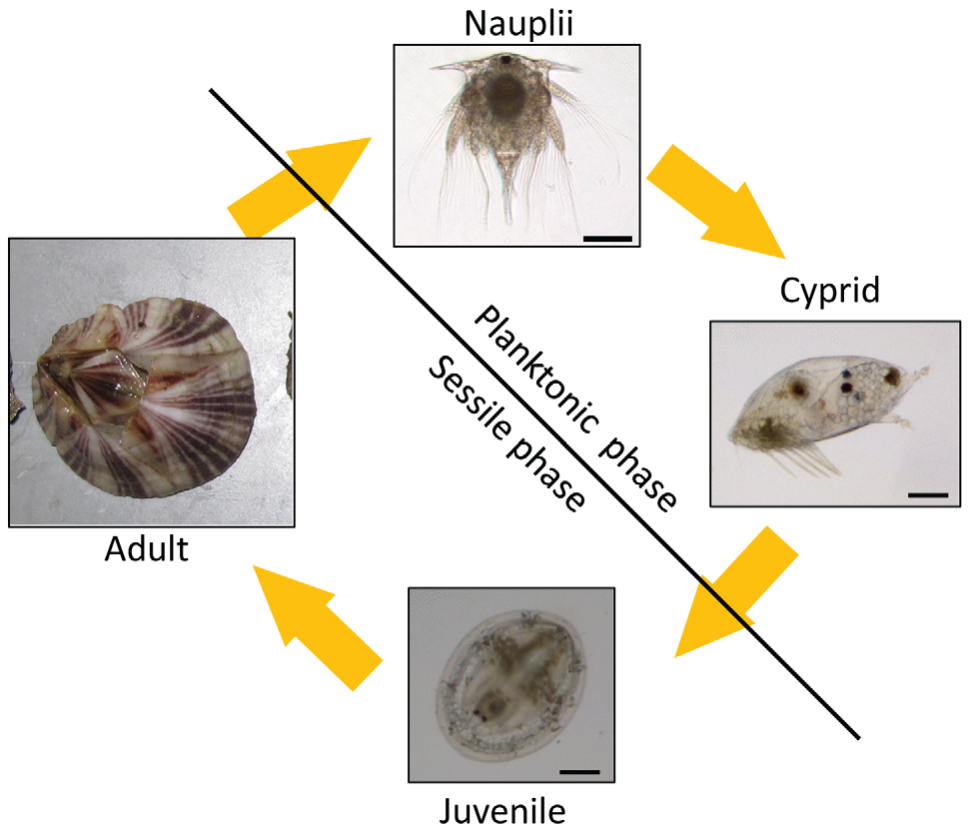
Life cycle of *Amphibalanus amphitrite*. The life cycle of barnacle is composed of two distinct phases: planktonic phase and sessile phase. The planktonic phase includes six feeding nauplius stages and one non-feeding cyprid stage; the sessile phase includes a juvenile and adult stages. Scale bar: 100 µm.

*Amphibalanus amphitrite*, an inter-tide marine organism, is a common fouling animal found all over the world. However, no effective antifouling measure has been found that is able to resolve this problem. Therefore, understanding how the barnacles glue themselves to substrata is essential. The barnacle is a bi-phase crustacean with a planktonic phase and a sessile phase (Fig. 1). The planktonic phase is composed of six feeding nauplius stages and one non-feeding cyprid stage. Therefore, the cyprid stage is critical for the barnacle from planktonic to sessile phase. The cyprid explores various surfaces using a pair of antennules. Once a site is selected, the cyprid releases adhesive called cement that it uses to glue itself onto the substratum. It then metamorphoses into a juvenile. As the juvenile matures into an adult the cement between the barnacle and the substratum becomes much stronger.

Proteins are the main component of the cement by weight, accounting for approximately 90% of the cement released from the barnacle [1], [2], The rest is a mixture of carbohydrate, ash and trace amounts of lipid [3]. Just above the compound eyes in a cyprid lies a pair of cement glands. This glandular structure within the larval body is where cement protein synthesis occurs. The cement gland has two secretory cell types: α and β cells [4]. The α secretory cells are columnar cells, about 65 µm tall and 20 µm wide with large basal nuclei, characterized by the presence of numerous round granules in the supranuclear cytoplasm [4]. The β secretory cells are rounded cells about 30 µm in diameter. Their cytoplasm is full of membrane bound secretory vacuoles [4]. The density of particles in α cells is much higher than in β cells when examined by electron microscopy [4].

According to the producing condition, barnacle cement is grouped into primary cement and secondary cement. The barnacle generates primary cement when it attaches to a substratum and produces secondary cement when free from a substratum [5]. The two types of cement share a similar amino acid composition. Peptide mapping of the cement proteins by SDS-PAGE after cyanogen bromide treatment show that they share the same protein components in *B. perforates*, *B. crenatus*, and *B. balanoides*. [3]. In addition, it has been reported that secondary cement enables barnacles to reattach to a substratum [5], [6]. This evidence indicates that the primary and secondary cement are essentially the same. However, little was known about the characteristics and function of cement proteins until several cement proteins were indentified from the adult barnacle [7]. More than ten cement proteins from *Megabalanus rosa* have been detected with SDS-PAGE. Five have been identified, including three large cement proteins: Mrcp100k, Mrcp68k, and Mrcp52k [8], and two smaller proteins: Mrcp19k and Mrcp20k [9], [10]. No homologues of these cement proteins were found in published databases.

Mrcp20k, defined by its molecular size, was isolated by reversed phase HPLC (high- pressure liquid chromatography) from the urea fraction of primary cement from *Megabarnalus rosa* [10]. In both primary and secondary cement, Mrcp20k appeared as a single band on SDS-PAGE in non-reducing conditions indicating a monomeric functional unit and a lack of disulfide bond formation in the Mrcp20k in the natural cement [11]. Mrcp20k was spotted in the basal plate and not the peripheral shell. This supports the hypothesis that Mrcp20k is a cement protein [11]. Mrcp20k consists of 202 amino acids, including a 19 amino acid signal sequence and the whole sequence of Mrcp20k is characterized by multiple repeated Cys domains. Moreover, Mrcp20k contains abundant charged amino acids including Asp (11.5%), Glu (10.4%) and His (10.4%) [10]. *Fistulobalanus albicostatus* has a homologue of Mrcp20k that is 125 amino acids long and shares four repeated Cys domains with Mrcp-20k. The first 20 amino acids were thought to compose a signal peptide [11].

It has been known that the cement secreted from a cyprid during exploring is temporary; however, the cement secreted by an adult is hard, which could permanently glue itself to the substratum. This phenomenon suggests the content or property of the cement is different between a cyprid and an adult. Two homologues of Mrcp-20k were found in our *A. amphitrite* transcriptome [12]. However, the function and characteristics of these two homologues are still unknown. In this study, we carried out a series of experiments to characterize the two homologues of Mrcp-20k in *A. amphitrite* and compare them between cyprids and adults.

## Results

### Characterization of the primary sequences of the Mrcp20k homologues in the barnacle, *Amphibalanus amphitrite*

**Figure 2:**
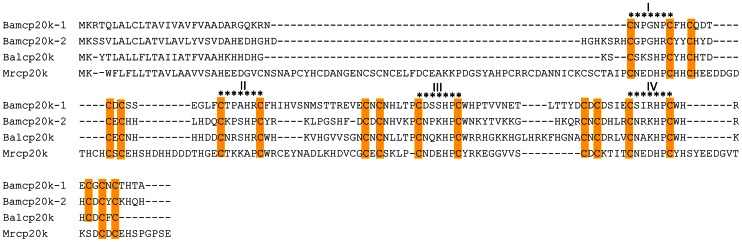
Alignment of the homologues of cp20k. *Amphibalanus amphitrite* cp20k-1 (Bamcp20k-1, JX826508) and cp20k-2 (Bamcp20k-2, JX826509) together with *Fistulobalanus albicostatus* cp20k (Balcp20k, BAF96022.1) and *Megabalanus rosa* cp20k (Mr cp20k, BAB18762.1) were aligned with CLUSTAL W software. Repeated Cys residues were highlighted in yellow. The motif of Cys-Xaa-Xaa-Xaa-Xaa-Xaa-Cys was indicated by asterisk. Xaa: any residue.

**Figure 3:**
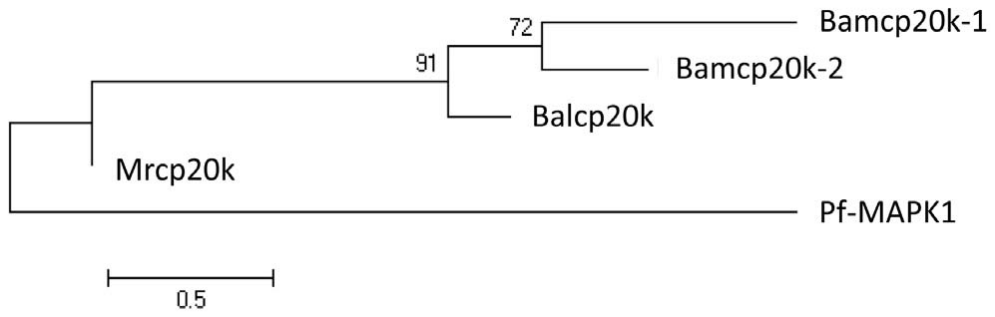
Phylogenetic tree analysis of cp20k. The full-length of the amino acid sequences were used in an alignment for phylogenetic tree calculations. The numbers indicate the occurrences of a branch point over 500 replications. The scale bar indicates the phylogenetic distances. Pf-MAPK1 was used as an out group protein.

Two homologues of Mrcp20k were found in our *A. amphitrite* transcriptome. To characterize these two homologues, the full-length coding sequences were cloned and named Bamcp20k-1 (JX826508) and Bamcp20k-2 (JX826509). Using the CLUSTAL W program, both Bamcp20k-1 and Bamcp20k-2 were aligned with the other two cp20k proteins from *Megabalanus rosa* and *Fistulobalanus albicostatus*, labeled as Mrcp20k and Balcp20k. Bamcp20k-1 and Bamcp20k-2 share multiple Cys domains with Mrcp20k and Balcp20k including four Cys-Xaa-Xaa-Xaa-Xaa-Xaa-Cys domains, five Cys-Xaa-Cys domains and one Cys-Xaa-Xaa-Cys domain (Fig. 2). Although the amino acids in the Xaa position were not conserved, charged amino acids were common. Phylogenetic tree analysis showed that Bamcp20k-1 was clustered with Bamcp20k-2. These two Bamcp20k proteins were more closely related to *Fistulobalanus albicostatus* than to *Megabalanus rosa* (Fig. 3).

Analysis of amino acid composition showed an abundance of Cys residues with more than 14% in the four cp20k proteins analyzed (Table 1). In Mrcp20k, Asp is another abundant residue at 10.4% and in Bamcp20k-1, Thr is abundant at 10.1%. For both Bamcp20k-2 and Balcp20k the most common amino acid is His at 16.8% (Table 1). The proportion of the residues mentioned above was significantly higher than standard amino acid compositions [13]. Bamcp20k-1, Bamcp20k-2, and Balcp20k appeared to have similar molecular masses, approximately 15kDa, roughly 7kDa smaller than Mrcp20k (Table 2). The difference in molecular size between Mrcp20k and other cp20k proteins is due to several missing repeated Cys domains (Fig. 2). Bamcp20k-1 and Bamcp20k-2 shared a similar primary sequence and molecular weight, but displayed very different PI (isoelectric point) values (Table 2). Bamcp20k-1 had an alkaline PI value of about 8.7. Whereas, Bamcp20k-2 had an acidic PI value of about 6.2 (Table 2).

**Table 1:** Amino acid composition of the cp20k homologues from three species.

Mrcp20k		Balcp20k		Bamcp20k-1		Bamcp20k-2	
AA	Per (%)	AA	Per (%)	AA	Per (%)	AA	Pe (%)
Cys (C)	15.8	His (H)	16.8	Cys (C)	14.7	His (H)	16.8
Asp (D)	10.4	Cys (C)	14.4	Thr (T)	10.1	Cys (C)	14.5
Glu (E)	9.4	Lys (K)	8.8	His (H)	7.8	Lys (K)	9.2
His (H)	9.4	Asn (N)	7.2	Ala (A)	6.2	Asp (D)	6.9
Ser (S)	6.4	Leu (L)	6.4	Val (V)	6.2	Leu (L)	6.1
Ala (A)	5.9	Ala (A)	5.6	Asn (N)	6.2	Pro (P)	6.1
Asn (N)	5.4	Arg (R)	4.8	Ser (S)	6.2	Arg (R)	5.3
Lys (K)	5.4	Asp (D)	4.8	Arg (R)	5.4	Tyr (Y)	5.3
Pro (P)	5.4	Gly (G)	4.8	Pro (P)	5.4	Val (V)	5.3
Gly (G)	5	Ser (S)	4	Asp (D)	5.4	Gly (G)	4.6
Thr (T)	4.5	Thr (T)	4	Glu (E)	4.7	Ser (S)	4.6
Val (V)	4	Val (V)	4	Leu (L)	4.7	Asn (N)	3.8
Leu (L)	3.5	Phe (F)	3.2	Ile (I)	3.1	Ala (A)	3.1
Tyr (Y)	3	Pro (P)	3.2	Phe (F)	3.1	Gln (Q)	2.3
Arg (R)	2	Trp (W)	2.4	Gly (G)	3.1	Glu (E)	1.5
Ile (I)	1.5	Ile (I)	1.6	Gln (Q)	2.3	Thr (T)	1.5
Phe (F)	1.5	Tyr (Y)	1.6	Met (M)	1.6	Trp (W)	1.5
Trp (W)	1	Gln (Q)	0.8	Trp (W)	1.6	Met (M)	0.8
Met (M)	0.5	Glu (E)	0.8	Lys (K)	1.6	Phe (F)	0.8
Gln (Q)	0	Met (M)	0.8	Tyr (Y)	0.8	Ile (I)	0
Pyl (O)	0	Pyl (O)	0	Pyl (O)	0	Pyl (O)	0
Sec (U)	0	Sec (U)	0	Sec (U)	0	Sec (U)	0

Amino acid composition of the homologues of cp20k was analyzed from *Amphibalanus amphitrite* cp20k-1 (Bamcp20k-1, JX826508), cp20k-2 (Bamcp20k-2, JX826509), *Fistulobalanus albicostatus* cp20k (Balcp20k, BAF96022.1), and *Megabalanus rosa* cp20k (Mrcp20k, BAB18762.1). The percentage of each amino acid is shown in the table. AA: amino acid; Per: percentage.

**Table 2:** Mass and PI of the homologues of cp20k from three barnacle species.

	Mrcp20k	Balcp20k	Bamcp20-1	Bamcp20-2
Mass (Da)	22466.57	14477.62	15395.57	14375.26
PI	4.95	8.91	8.68	6.16

Mass and PI of the four homologues were shown from *Amphibalanus amphitrite* cp20k-1 (Bamcp20k-1, JX826508), cp20k-2 (Bamcp20k-2, JX826509), *Fistulobalanus albicostatus* cp20k (Balcp20k, BAF96022.1) and *Megabalanus rosa* cp20k (Mrcp20k, BAB18762.1).

### Localizations of Bamcp20k-1 and Bamcp20k-2 in cyprid

**Figure 4:**
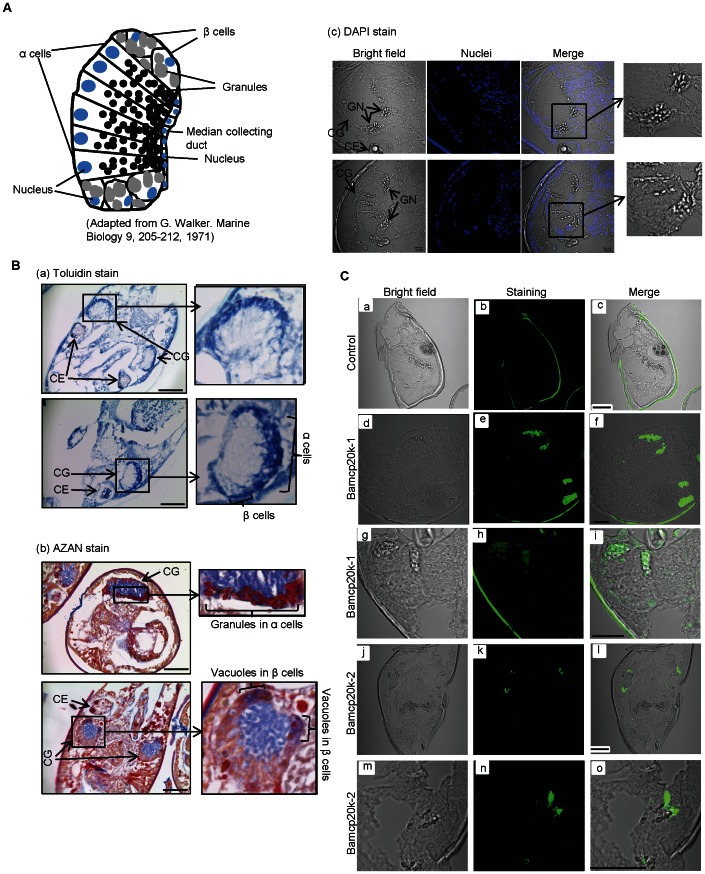
Differential localizations of Bamcp20k-1 and Bamcp20k-2 in cyprid cement glands. (A) The diagram shows the composition of a cement gland in *Balanus balanoides*. Two types of cement cells (α and β) were shown in the cement glands. (B) Cyprids were fixed in 4% PFA and then sectioned after embedding in wax. A section of 4 µm from *Amphibalanus Amphitrite* was shown. (a) Toluidin stain, Scale bar: 50 µm; (b) AZAN stain, Scale bar: 50 µm; (c) DAPI stain. Nuclei were stained with DAPI. Scale bar: 10 µm. CE: compound eye; CG: cement gland; GN: granules; NC: nuclei. (C) Bamcp20k-1 and Bamcp20k-2 were localized at different cement granules. Sections were immunostained with antibodies against Bamcp20k-1 and Bamcp20k-2. For (a) to (c), the sections stained only with secondary antibody were used as negative controls; for (d) to (i), the sections were stained with an antibody against Bamcp20k-1; for (j) to (o), the sections were stained with an antibody against Bamcp20k-2. Scale bar: 30 µm.

To investigate the function of Bamcp20k-1 and Bamcp20k-2 in *A. amphitrite*, their localizations within the cyprid were examined. Following fixation and sectioning described in the methods, the cyprids were immunostained with antibodies against Bamcp20k-1 and Bamcp20k-2. α and β secretory cells in the cyprid cement gland were identified morphologically. α cells were columnar with high-density granules. The adjacent β cells had a much lower granule density (Fig. 4A and B) [4], which made it difficult to identify them under the light microscope. By AZAN stain, the granules in α cells appeared red, meanwhile, the vacuoles in β cells were blue (Fig. 4B), which was consistent with the previous report [4]. The results showed that Bamcp20k-1 was localized in the α secretory cells within the paired cement glands. Higher magnification images further showed that Bamcp20k-1 was exclusively localized in the individual granules of α secretory cells (Fig. 4C). Bamcp20k-2 localized near the α secretory cells, most likely within the β secretory cells (Fig. 4C). The control image, in which only secondary antibody was used, showed no significant localization pattern (Fig. 4C).

### Adult Bamcp20k-1 and Bamcp20k-2 appeared to have different solubility in PBS buffer

**Figure 5:**
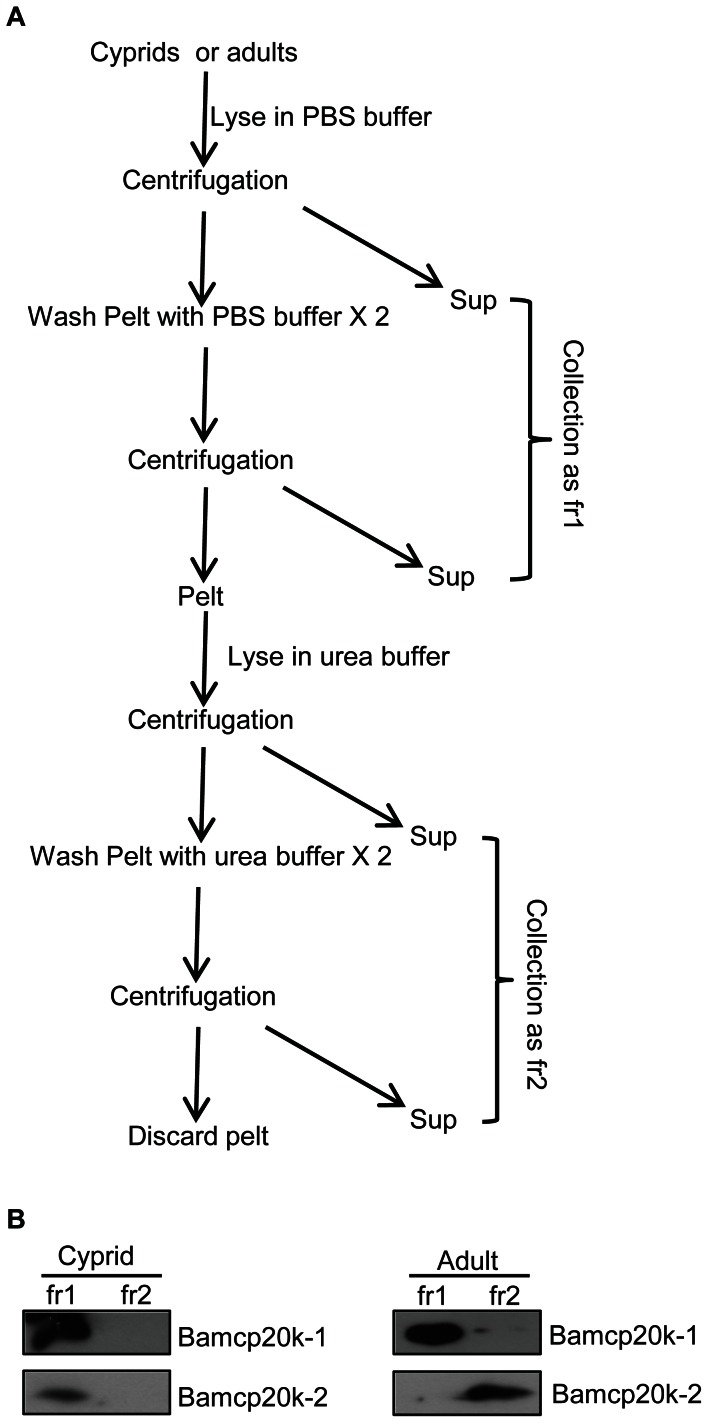
Adult Bamcp20k-2 was insoluble in PBS. (A) PBS and urea soluble fractions were prepared from cyprids and adults. Cyprids or adults were extracted in PBS. After centrifugation, the supernatant was collected and the pellet was washed with the same buffer. The supernatant from each centrifugation was collected and labeled as fr1. The pellet from the final centrifugation was sequentially extracted in urea buffer. After a series of successive centrifugations mentioned above, the supernatant were collected and labeled as fr2. Fr: fraction; sup: supernatant; pelt: pellet. (B) Equal proportions from each fraction were collected for Western blot with the antibodies against Bamcp20k-1 and Bamcp20k-2. Fr: fraction.

The cyprid and adult barnacle proteins were sequentially extracted in PBS and urea buffer as described in the methods section and displayed in figure 5A. Equal proportions from the two fractions were collected for blotting against Bamcp20k-1 and Bamcp20k-2. Results revealed that cyprid Bamcp20k-1 and Bamcp20k-2 were soluble in PBS buffer. However, adult Bamcp20k-1 was enriched in PBS, while adult Bamcp20k-2 was only found in the urea fraction (Fig. 5B).

### Bamcp20k-1 and Bamcp20k-2 have differential distributions in adult barnacles

**Figure 6:**
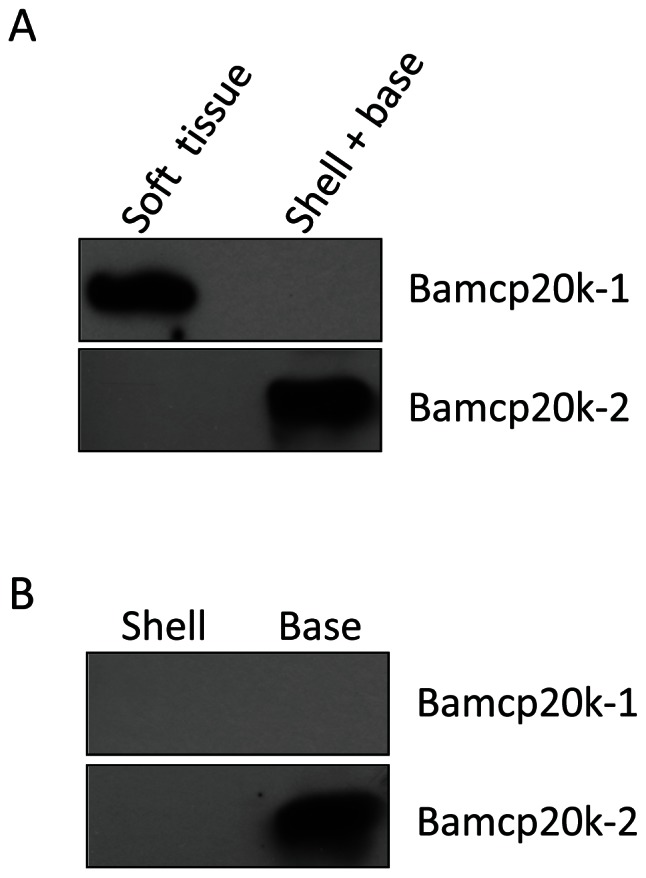
Bamcp20k-2 was enriched in the basal plate. (A) An adult barnacle was divided into two portions: the soft tissue and the shell with base. These two portions were extracted in urea buffer. Equal proportions from each fraction were collected for Western blot against Bamcp20k-1 and Bamcp20k-2. (B) Equal amounts of extract (60 µg) from shell and base were collected and blotted with the antibodies against Bamcp20k-1 and cp20k-2. Fr: fraction.

The distribution patterns of adult Bamcp20k-1 and Bamcp20k-2 in adult barnacles were examined due to their differential solubility. Each adult barnacle was divided into soft tissue and shell with base parts. Then the two samples were extracted in urea buffer. Equal proportions from the two parts were collected and blotted against Bamcp20k-1 and Bamcp20k-2. The results showed that Bamcp20k-1 was present in soft tissue but could not be easily observed in the shell with base portion in the Western blot. However, Bamcp20k-2 was detected the shell with base portion (Fig. 6A), and particularly highly expressed in the base (Fig. 6B).

### DTT promoted Bamcp20k-2 solubility

**Figure 7:**
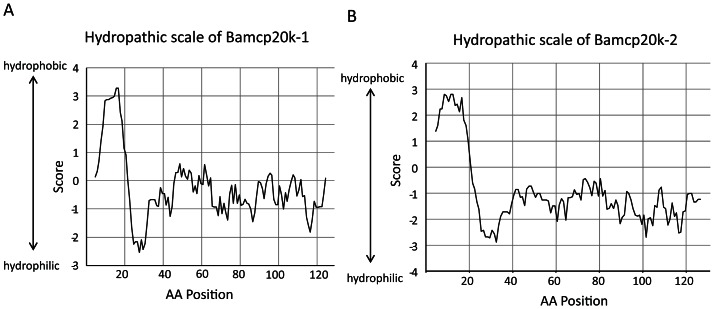
Hydrophobicity analysis of Bamcp20k-1 and cp20k-2. (A) and (B) amino acids derived from the sequences of Bamcp20k-1 and cp20k-2 were analyzed for hydrophobicity property. The x-axis indicates the amino acid position; y-axis indicates the score of hydrophilicity and hydrophobicity.

**Figure 8:**
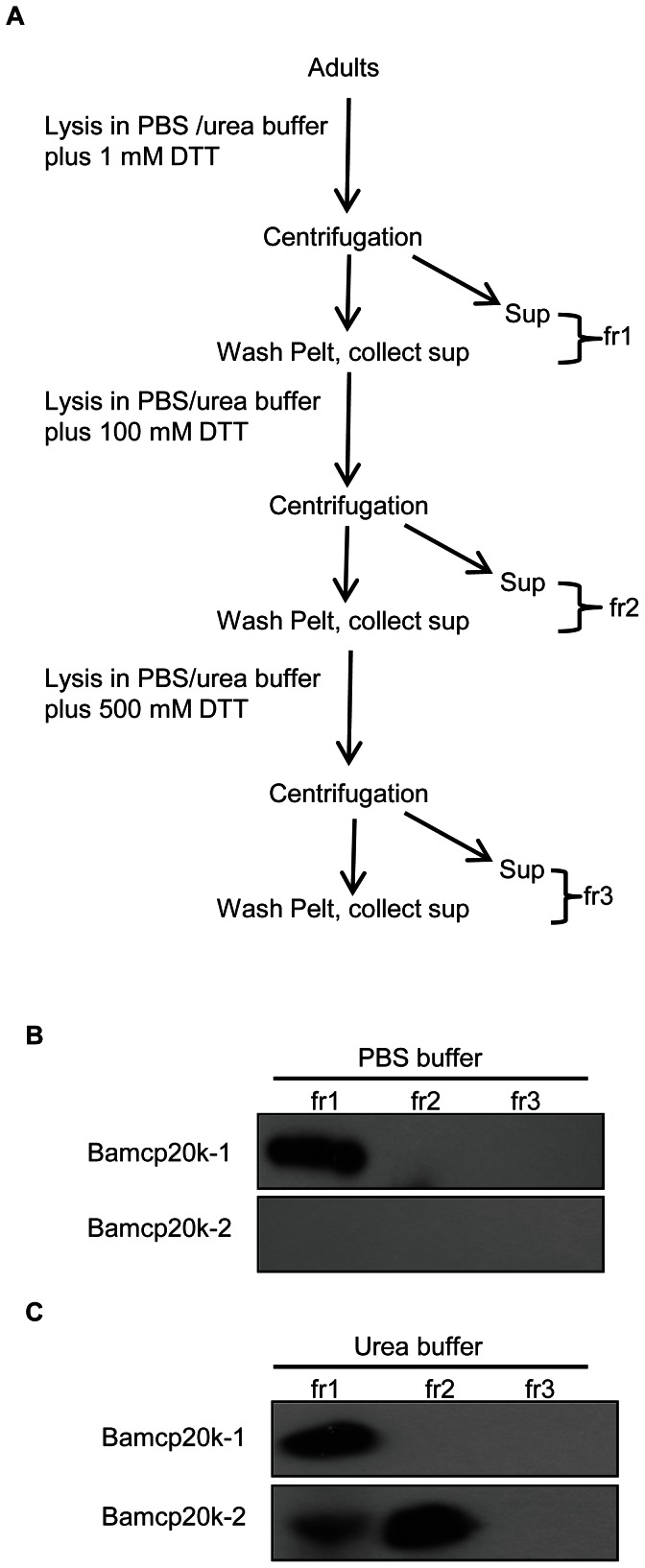
Solubility of adult Bamcp20k-2 was dependent on DTT concentration. (A) and (B) Adult barnacles were homogenized in PBS or urea buffer with different concentration of DTT, as mentioned in the methods section. Equal proportions from each fraction were collected for Western blot against Bamcp20k-1 and Bamcp20k-2. Fr: fraction.

To further investigate the factors that affect Bamcp20k-2 solubility, hydropathic scales of both Bamcp20k-1 and Bamcp20k-2 were calculated. The results showed that Bamcp20k-1 and Bamcp20k-2 shared a similar pattern of hydrophobicity. The first 20 amino acids were hydrophobic in both Bamcp20k-1 and Bamcp20k-2, ranging from 0 to 4 in score. The remaining amino acids were mostly hydrophilic ranging from 0 to −3. This is especially true for Bamcp20k-2 where the scores ranged from −1 to −3 (Fig. 7A and B). The first 20 amino acids were thought to be the signal peptide. Analysis of the hydropathic scales indicates that the insolubility of the adult Bamcp20k-2 in PBS buffer is not due to the hydrophobic amino acids. The adult barnacles were sequentially extracted in PBS or urea buffer containing 1 mM, 100 mM, or 500 mM DTT (Fig. 8A). The results showed that adult Bamcp20k-2 was insoluble in PBS buffer even with 500 mM DTT (Fig. 8B). While, a small amount of adult Bamcp20k-2 dissolved in urea buffer containing 1 mM DTT, the majority of adult Bamcp20k-2 dissolved in urea buffer containing 100 mM DTT. No additional adult Bamcp20k-2 could be extracted with urea buffer containing 500 mM DTT (Fig. 8C). In contrast, the adult Bamcp20k-1 dissolved in both PBS and urea buffer containing as little as 1 mM DTT (Fig. 8B and C).

## Discussion

In this study, we cloned two Mrcp20k homologues from *Amphibalanus amphitrite*, Bamcp20k-1 and Bamcp20k-2. The two homologues from adult barnacles displayed differences in their spatial distribution and solubility. Bamcp20k-1 was enriched in the soft tissue and soluble in PBS buffer. Whereas, Bamcp20-2 was predominately present in the base plate and was insoluble in PBS even in the presence of 500 mM DTT. The solubility of adult Bamcp20k-2 was dependent on DTT concentration in the presence of the denaturing reagent urea. In the cyprid cement gland, Bamcp20k-1 localized at the granules of the α secretory cells, while Bamcp20k-2 localized at the β secretory cells. The differential solubility and localization of Bamcp20k-1 and Bamcp20k-2 suggests that they have different roles in barnacle cementing.

Cement proteins are the primary component in barnacle cement and they play a key role in barnacle adhesion. Therefore, studying cement proteins is essential in trying to understand the mechanism of barnacle settlement. Several cement proteins have been identified from the secondary cement of *M. rosa*, including three large cement proteins Mrcp100k, Mrcp68k, and Mrcp52k; and two smaller proteins Mrcp20k and Mrcp19k [7]. One homologue of Mrcp20k has been also isolated from *F. albicostatus*, Balcp20k [11]. When Balcp20k and Mrcp20k were aligned with Bamcp20k-1 and Bamcp20k-2 several repeated Cys motifs were shared (Fig. 1). Moreover, Cys comprised a high percentage of the amino acids, 14%, in both Bamcp20k-1 and Bamcp20k-2. The high percentage of Cys in both Bamcp20k-1 and Bamcp20k-2 is consistent with Mrcp20k and Balcp20k (Table.1.) [10], [11]. However, the function of Cys in these sequences is unknown. Conversely, Cys comprises only 1% of Mrcp19k and Mrcp100k [2], [9]. This feature of the cp20k homologues suggests that it has a distinct role from other known cement proteins.

In this study, the solubility of the adult Bamcp20k-2 was dependent upon DTT concentration (Fig. 7B), suggesting the formation of disulfide bonds in Bamcp20k-2 function. The predicted molecular mass of Balcp20k led us to assume that disulfide bonds were formed [11]. However, Mrcp20k could be completely extracted from both primary and secondary cement with guanidine hydrochloride buffer without a reducing reagent, indicating that Mrcp20k is not covalently crosslinked by disulfide bonds in natural cement despite the high percentage of Cys residues [10], [11]. This evidence suggests that the Cys residue is necessary but not sufficient to form disulfide bonds in cement proteins.

Bamcp20k-1 and Bamcp20k-2 were distributed differently in adult barnacle tissue. Bamcp20k-1 was primarily localized in the soft tissue and soluble in PBS buffer while Bamcp20k-2 was predominantly found in the base plate and was mostly insoluble in PBS. The insolubility of Bamcp20k-2 is thought to be due to crosslinking by disulfide bonding which may be triggered by seawater. The effect of a reducing reagent on barnacle attachment was confirmed in the detachment experiment using DTT treatment [8]. Bamcp20k-1 and Bamcp20k-2 have a conserved primary sequence with 35% identity and 52% similarity at the amino acid level (data not shown). Hydrophobicity analysis showed that Bamcp20k-1 and Bamcp20k-2 also share a similar hydrophobic pattern (Fig. 7). The first 20 amino acids are more hydrophobic, suggesting that this is the signal peptide, which is consistent with Mrcp20k [14]. However, the PI values of Bamcp20k-1 and Bamcp20k-2 are very different. Bamcp20k-1 is alkaline and Bamcp20k-2 is acidic, which may explain why disulfide bond formation in Bamcp20k-2 but not in Bamcp20k-1.

The cement glands are the location of cement proteins synthesis. There are two types of secretory cells in the cement glands: α and β secretory cells, which can differ morphologically. The granules in α secretory cells stained red with azan and were histochemically positive for proteins, phenols, and the enzyme polyphenol oxidase. The vacuoles in β secretory cells stained blue with azan, but were only histochemically positive under bromophenol blue examination [4]. These phenomena are consistent with another histochemical study of *B. eburneus* cypris larval cement; that show that proteins were the main component of cement but a small amount of lipid might also be present [15]. The proteinaceous nature of cyprid cement was also demonstrated in *B. crenatus*, which confirmed the presence of phenolic amino acids [16]. In our study, immunostaining revealed that Bamcp20k-1 and Bamcp20k-2 localized at α and β secretory cells, in the cement glands respectively (Fig. 4), which indicates that the granules in α and β secretory cells contains different protein contents. This finding is consistent with previous reports. The contents of the granules in α and β secretory cells were determined by staining with various dyes [4]. Bamcp20k-1 and Bamcp20k-2 localized at different secretory cells, suggesting differential regulations for their release during the barnacle cementing process. A previous study in *B. improvisus* cyprid showed that the neurotransmitter dopamine stimulated granule secretion [17]. While only one type of secretory cells was observed in that study. The results indicated that cement release from the cement gland was regulated by a neurotransmitter. Therefore, it is possible that release of Bamcp20k-1 and Bamcp20k-2 from α and β secretory cells in the cement gland of *A. amphitrite* is regulated by different neurotransmitters. During substrate exploration, cyprids release temporary cement through the paired antennules for reversible adhesion, whereas adults release permanent cement throughout their life. The differences between temporary and permanent cement are unknown, but temporary cement is most likely not as strong or as hard as permanent cement. Adults glue themselves permanently onto a surface; however, cyprids do not. As cement is primarily composed of proteins, it is conceivable that the cyprid and adult barnacles release different cement proteins when necessary. It is not clear whether the cement proteins secreted by cyprid and adults are functionally related, but the presence of Bamcp20k-1 and Bamcp20k-2 in both cyprid and adult cement suggests the development or maturation of the cement gland from the cyprids to the adult form. The development of the cement gland throughout the barnacle lifecycle requires further investigation.

Mrcp100k was the major component in both the primary and secondary cement from *M. rosa*. It was soluble in guanidine hydrochloride with the reducing reagent DTT, but insoluble without DTT [8]. We found that a homologue of Mrcp100k in *A. amphitrite* localized in the β secretory cells of the cyprid cement glands and that was primarily present in the adult base plate (data not shown). This property of Bamcp100k is quite similar to Bamcp20k-2, which also localized in the β secretory cells and was predominantly present in the base plate (Fig. 8). Mrcp19k was only present in the base portion [9], indicating that it has a unique role in barnacle cementing. The homologue of Mrcp19k was also found in *A. amphitrite* [12]. Therefore, if all of the cement proteins, which were localized at the β secretory cells in the cyprid cement gland, were found predominantly in the adult basal plate. It is possible that the β secretory cells develop into the major secretory cells in the adult cement glands due to the large demand in the adult stage of the barancle. Previous reports demonstrated that only one type of secretory cell was observed in the adult cement glands [18], [19]. It is possible that this secretory cell type was derived from the β secretory cells in the cyprids and that the α secretory cells in cyprid cement glands had shrunk making them difficult to be detected.

Cement proteins were thought to play a critical role during barnacle larval settlement. When exploration for finding a suitable site, the larva releases cement proteins to temporarily glue itself to the substratum and when metamorphosis into a juvenile and then growth up into an adult, it releases cement proteins to permanently maintain itself to the substratum. In contrast, the larvae from the close relatives of barnacle including Ascothoracida and Facetotecta, which lack cement glands, attach mechanically [22]. The adults of Facetotecta, which have never been seen by people, are little known. And the adults of Ascothoracida are a fundamentally non-sessile group in contrast to Cirripedia. Therefore, it is possible that the cement proteins are essential for the barnacle permanent attachment to the substratum.

In this study, two homologues of Mrcp20k in *A. amphitrite* were isolated, cloned and characterized. These homologues shared conserved repeated Cys domains. Both Bamcp20k-1 and Bamcp20k-2 localized at the granules in the cyprid cement glands but within different secretory cell types. Though the two homologues are highly conserved, they have different solubility and anatomical distributions, suggesting that they have distinctive roles in the barnacle cementing process.

## Materials and Methods

### Ethics Statement

Adult barnacles, *Amphibalanus amphitrite* were collected from populations growing on the concrete poles at Pak Sha Wan in Hong Kong (22°21′45″N, 114°15′35″E). The barnacles, are neither an endangered nor protected animal, but are a common biofouling species in marine environments. The dock does not belong to a national park, protected area, nor is it privately owned. Thus, the adult barnacles were collected from the field without needing a specific permit and no endangered or protected species were affected.

### Larval culture

Larvae were released from the adult barnacles of *Amphibalanus amphitrite* immediately after collection from the field and cultured in the laboratory according to the procedures described in He et al. (2012) [20]. Briefly, the released larvae were maintained in 0.22 µm filtered seawater (FSW) with a density of 1 larva ml^−1^ at 28°C. They were fed with *Chaetoceros gracilis* and their water was changed daily. Nauplii metamorphose into cyprids on day 4 after six molts; five molts from nauplius I to nauplius V and one molt from nauplius V to cyprids.

### Cloning of target genes

RNA extraction and cDNA preparation were carried out following the procedures described in He et al (2012) [20]. Briefly, total RNA was extracted using the TRIzol reagent according to the manufacturer's instructions (Invitrogen, USA). Subsequently, cDNA was synthesized from the total RNA using M-MLV reverse transcriptase (Ambion, USA) using oligo dT primers [21]. Based on the partial sequences of the two homologues of Mrcp20k in the barnacle transcriptome database, specific primers were designed and used for the rapid amplification of cDNA ends (RACE) reactions. This is done to obtain the full-length coding sequences of the two homologues [20] that were then confirmed by sequencing (BGI Company, China).

### Sequence alignment and phylogenetic analysis

Multiple sequence alignments were performed using the CLUSTAL W version 1.83 program and graphically displayed with the software BioEdit 7.0.0. The rooted phylogenetic tree was generated with aligned amino acid sequences by the Maximum Likelihood method in the MEGA software (version 5.05). The supporting degree for internal branches was further assessed by bootstrap analysis with 500 replications. ExPASy tools (http://www.expasy.org/tools/) were used to calculate the amino acid composition, molecular mass, and PI.

### Plasmid construction, recombinant protein over-expression, and antibody generation

Full-length sequences of the two homologues of Mrcp20k in *Amphibalanus amphitrite* were used to generate antibodies. These two homologues were cloned into pGEX4T and pet21b vectors. The recombinant GST and His_6_ tagged proteins were overexpressed in *E. coli* BL21 (DE3), then isolated according to the protocol described by He et al (2012) [20] which uses GST-Sepharose (Sigma, USA) or Ni^2+^ -nitrilotriacetic acid beads (Qiagen, USA). Antibody generation was performed according to the procedure in the same publication [20]. Briefly, His_6_ tagged recombinant proteins were used as an antigen to immunize New Zealand white rabbits with Freund's adjuvant (Sigma, USA). The pre-injection serum was used as negative control and the post-immune serum was collected after four injections. GST-tagged recombinant proteins were used as bait to isolate the antibody.

### Immunofluorescence imaging

The cyprids were collected within 24 hr after metamorphosis from nauplius and fixed with 4% paraformaldehyde (PFA) in phosphate-buffered saline (PBS) overnight at 4°C. Following progressive dehydration in ethanol solution (70%, 85%, 95% and 100%) for 30 min each, the cyprids were incubated with a mixture solution including 50% ethanol and 50% xylene (VMR, USA) for 30 min. After two additional incubations with pure xylene, the cyprids were embedded in prewarmed paraffin wax (Paraplast Plus, Kendall). Sections were cut with 4 µm thick and mounted onto glass cover slips. Rehydration was performed in a series of ethanol solution (100%, 90%, 75% and 35%) after drying at room temperature overnight. The sections were incubated in 10 mM citric acid at 95°C for 15 min before permeabilization with 0.2% Triton-X100 in PBS. After blocking with 5% bovine serum albumin (BSA) in PBS overnight at 4°C, the sections were subjected to immunostaining with antibodies against the two homologues of Mrcp20k overnight at 4°C. Primary and secondary antibodies were diluted in PBS containing 3% BSA at 1∶300 and 1∶1000 dilutions, respectively. Slides were washed with PBS between primary and secondary antibody incubations. Cyprid sections stained with anti-rabbit secondary antibody alone served as negative controls. The nuclei were stained with DAPI (Sigma, USA). Images were collected with a laser scanning confocal microscope (Zeiss, LSM 710, ZEN 2009 software, USA).

#### Toluidine blue stain

Cyprids were fixed and embedded as above described. After a series of rehydration, the sections with 4 µm were incubated with 0.5% toluidine blue (Fluka, USA) at room temperature for 30 min. Following the removal of toluidine blue, the sections were differentiated by 0.5% acetic acid for 1 min. After dehydration with absolute alcohol and clarity with xylene, the sections were mounted for observation using a light microscope (BX-51 compound microscope, Olympus) with DIC setting.

#### AZAN stain

Cyprid sections were prepared as above described and then the AZAN stain was performed according to the protocol provided by the company (Heidenhain's Aniline Blue-AZAN Stain Kit, American MasterTech, USA). Briefly, the sections were incubated with prewarmed Azocarmine B solution at 56°C for 15 min and then moved to room temperature for another 1 hr. After rinse with distilled water, the sections were differentiated with 0.1% Aniline alcohol solution until cytoplasm and nuclei were well defined. The differentiation was stopped with 1% Acetic alcohol solution. After the incubation with 5% Phosphotungstic acid for 1 hr at room temperature, the sections were incubated with Aniline blue solution for 1 hr at room temperature. Following a rinse with distilled water, dehydration with absolute alcohol and clarity with xylene were carried out and then mounted for observation using a light microscope (BX-51 compound microscope, Olympus) with DIC setting.

### Protein extraction and Western blot analysis

Cyprids were collected within one day after molting from nauplii and then lysed in PBS containing 1 mM dithiothreitol (DTT) plus protease and phosphatase inhibitors (Roche, Germany). After sonication (Branson Digital Sonicator 250, Danbury, CT), the samples were centrifuged at 20,000×g to pellet debris and supernatants were collected. The pellet was washed twice with lysis buffer and then centrifuged again. The supernatants were collected together and labeled as fraction 1. The pellet was subsequently extracted in urea buffer (8 M urea pH 7.5, 100 mM DTT, 1% sodium dodecyl sulfate (SDS) with protease and phosphatase inhibitors). After centrifugation, the pellet was washed twice with the same urea buffer and all of the supernatants were collected together as fraction 2. The adult extract was obtained by homogenization with the same buffer as above. Following the same steps, fraction 1 and fraction 2 were collected. Equal proportions of fraction1 and fraction 2 were subjected to SDS-PAGE and then transferred to a 0.22 µm-PVDF membrane (Millipore, USA) for Western blotting with the antibodies developed against the two homologues.

To investigate the distribution of the two homologues in adult barnacles, fresh adult barnacles were divided into soft tissue and shell with base parts. Briefly, the soft tissue was removed from the shell and then lysed with urea buffer (8 M urea pH 7.5, 500 mM DTT, 1% SDS, protease and phosphatase inhibitors). To avoid the contamination from soft tissue, the interior of the shell were thoroughly washed with the same buffer. The cleaned shell was then crushed with the same lysis buffer. Equal proportions of the two collected samples were taken to be blotted against the two homologues. To study the shell and base distribution, an equal amount of protein was collected from them both and used for blotting against the two homologues.

To examine the effect of DTT on protein solubility, fresh adult barnacles were sequentially lysed in PBS and urea buffer (8 M urea pH 7.5, 1% SDS, protease and phosphatase inhibitors) with increasing concentrations of DTT (1 mM, 100 mM and 500 mM). In brief, adult barnacles were lysed in PBS or urea buffer with 1 mM DTT, after washing with the same buffer, supernatants from each centrifugation were collected and labeled as fraction 1 and the pellets were lysed in PBS or urea buffer with 100 mM DTT. After the same washing steps, the supernatants were collected and labeled as fraction 2. The pellets were sequentially lysed in PBS or urea buffer with 500 mM DTT and the supernatants were collected as fraction 3. Equal proportions from each fraction were collected for blotting against the two homologues.

## Supporting Information

Figure S1 **The full patterns of Western Blots against Bamcp20k-1 and Bamcp20k-2.** Cyprids and adults were extracted in PBS buffer and then subjected to the SDS-PAGE gel. Bamcp20k-1 and Bamcp20k-2 were blotted by their antibodies, respectively.
(TIF)
