# Supplementary figures and images for: Characterization of Two 20kDa-Cement Protein (cp20k) Homologues in Amphibalanus amphitrite
Source: PLoS One. 2013 May 22;8(5):e64130. doi: 10.1371/journal.pone.0064130 (PMC3661472; doi:10.1371/journal.pone.0064130)

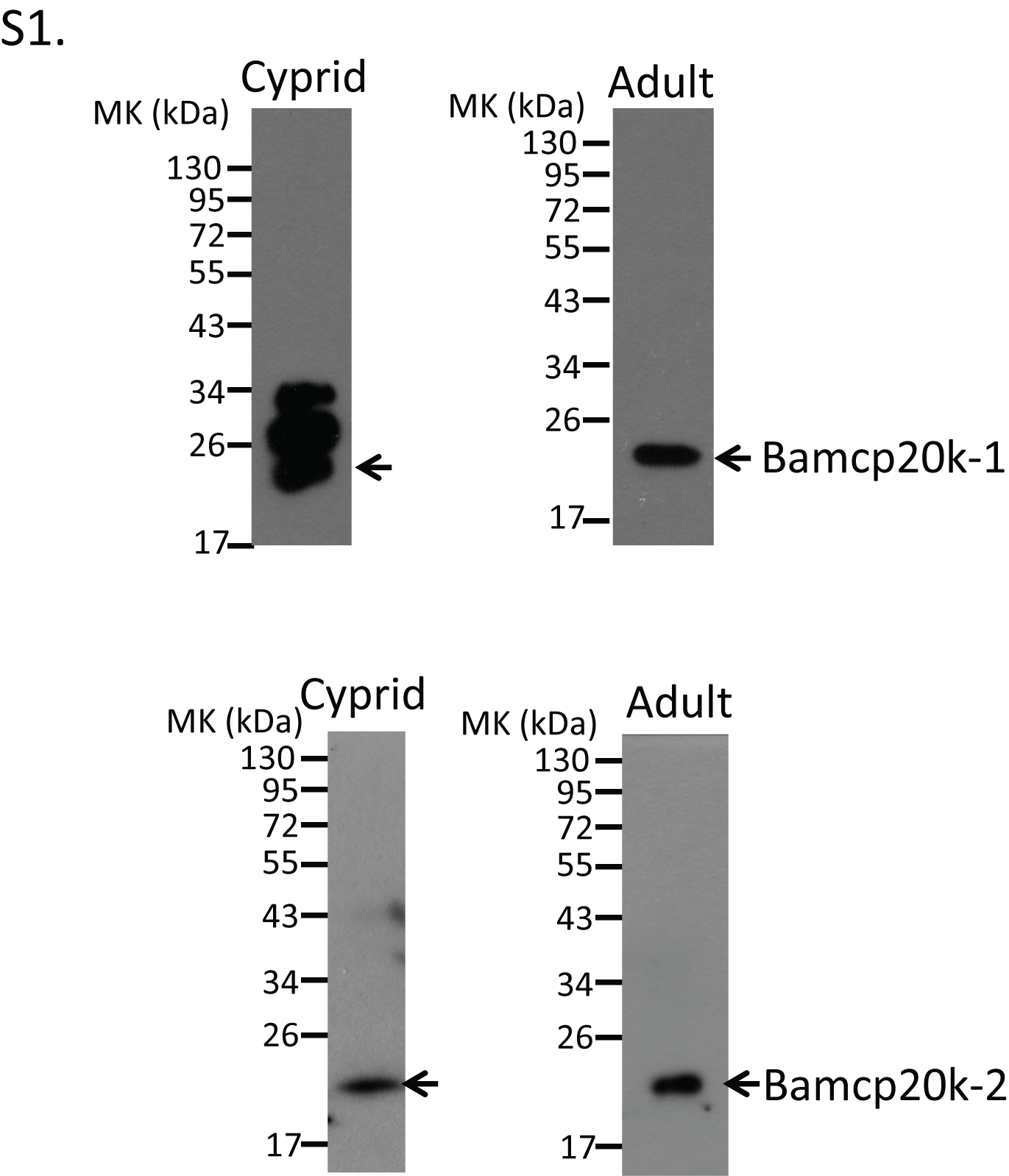

Supplement: Figure S1 — The full patterns of Western Blots against Bamcp20k-1 and Bamcp20k-2. Cyprids and adults were extracted in PBS buffer and then subjected to the SDS-PAGE gel. Bamcp20k-1 and Bamcp20k-2 were blotted by their antibodies, respectively. (TIF) [file pone.0064130.s001.tif]
